# Response

**DOI:** 10.1186/s13054-019-2458-x

**Published:** 2019-05-20

**Authors:** Huoyan Liang, Xianfei Ding, Tongwen Sun

**Affiliations:** General ICU, The First Affiliated Hospital of Zhengzhou University, Henan Key Laboratory of Critical Care Medicine, Zhengzhou, 450052 China

To the Editor:

We thank Dr. Ye and colleagues [1] for their interest in our article in Critical Care [2]. First, we agree with their view that diabetes covariates may affect the efficacy of preadmission metformin use in septic patients with diabetes. Although one retrospective large database review [3] reported that diabetes was not associated with adjusted 90-day mortality risk in critically ill patients admitted with sepsis, our study used adjusted ORs and 95% CIs, which were corrected for the diabetes covariate or which balanced the blood glucose variation between the metformin and non-metformin groups. When the unadjusted ORs and 95% CI data were extracted in one study [4], they showed negative results between metformin and non-metformin use. Therefore, neither the covariates of diabetes nor variations in blood glucose levels had significant effects on the outcomes of our study. Second, we acknowledge that we did not adequately discuss heterogeneity in our study, although all meta-analyses have varying degrees of heterogeneity. Therefore, the Simplified Acute Physiology Score II, Acute Physiology and Chronic Health Evaluation II (APACHE) II score, and the Sequential Organ Failure Assessment (SOFA) score baselines may not have been the factors that lead to the heterogeneity in our study. Third, we thank Dr. Ye and colleagues for pointing out our mistake in the number of included studies in the discussion section of our study. Finally, we believe that clinical trials will confirm the relationship between diabetes and mortality in patients with sepsis, and the studies to assess the association between metformin and mortality in septic patients with diabetes will be performed.
